# Hypertension in Young Asymptomatic University Students: Impact of Parental History, Physical Inactivity, and Diet

**DOI:** 10.7759/cureus.69615

**Published:** 2024-09-17

**Authors:** Savita Bansal, Kajasai Ragesvari, Manmeet Kaur

**Affiliations:** 1 Pathology, Manav Rachna Dental College, Faridabad, IND

**Keywords:** fast food, hypertension, parental history of hypertension, physical inactivity, university students

## Abstract

Introduction

Non-communicable diseases such as hypertension have emerged as a major public concern over the past two decades due to lifestyle changes. Patients with prehypertension have a greater risk of developing coronary artery disease, myocardial infarction, and stroke. Hypertension is a major modifiable risk factor for premature death worldwide. Evidence about the prevalence of hypertension and related variables, however, is sparse among university students in areas with limited resources. This study was conducted with an aim to evaluate the impact of parental history, physical inactivity, and diet on the blood pressure readings of university students.

Methodology

For this study, 337 university students aged between 18 and 30 years (102 males and 235 females) were randomly selected. The data was collected through a structured questionnaire, which gathered information about the lifestyle habits of the participating students. Trained students measured the participant's blood pressure according to a standardized procedure.

Results

Of the total participants, 50.7% (n= 171) were normotensive, 36.8% (n= 124) were prehypertensive, and 12.5% (n= 42) were hypertensive. Males were more prone to developing prehypertension (54.9%) while females were more likely to develop hypertension (12.7%). The consumption of sugared beverages and Western fast food showed a statistically significant relationship with increased blood pressure. A significant relationship was also seen between the duration of exercise and hypertension (p-value =0.007)

Conclusion

Hypertension and prehypertension are common among university students. The risk factors for these conditions include a positive family history, being male, increased intake of fast food and sugar-sweetened beverages, and reduced duration of regular exercise. These findings emphasize the significance of implementing targeted health education programs aimed at fostering healthy lifestyle practices among young adults.

## Introduction

With the advent of globalization and digitalization, there has been a significant lifestyle shift, which has resulted in an increase in non-communicable diseases such as hypertension, diabetes, and cardiovascular conditions. Hypertension has emerged as a major public health issue in the past two decades, causing almost 71% of all deaths globally [1]. Moreover, hypertension is registering an increasing trend in young adults. In 2019, the Global Burden of Disease (GBD) reported that hypertension was responsible for about 10.8 million deaths, which is comparable to 9.3% disability-adjusted life years (DALYs). It was also speculated that approximately 1.56 billion people will suffer from hypertension by the year 2025 [1]. Hypertension is defined as persistently increased blood pressure, with a systolic value of 140 mmHg or above and a diastolic value of 90 mmHg or more, according to the Eighth Joint National Committee (JNC 8) guidelines. It arises from systemic changes affecting peripheral blood flow and pathological mechanisms, including elevated cardiac output and total peripheral vascular resistance [2]. A study reported the age- and sex-standardized prevalence rates of hypertension according to the JNC 8 and the 2017 American College of Cardiology (ACC)/American Heart Association (AHA) guidelines, which were 37.08% and 58.52% for the total population [3]. Furthermore, the prevalence rate respectively for men was 37.86% and 60.85%, while for females, it was 36.28% and 56.14%, respectively [3]. Patients with prehypertension have a 1.7 times greater risk of developing coronary artery disease and 3.5 times more risk of myocardial infarction. Prehypertension is also associated with an increased risk of premature death as stated by the Seventh Report of the Joint National Committee (JNC 7) [4].

From 1990 to 2019, the number of hypertension cases has doubled, with two-thirds occurring in low- and middle-income countries due to a higher prevalence of risk factors in these populations [5]. Recently, there has been an age shift, affecting younger age groups. Studies show that 7.4% of the university students in Ethiopia, 7% in Kuwait, 9.3% in Saudi Arabia, 19.3% in Uganda and 8.1% in the Gulf region have hypertension [6]. Additionally, 85-90% of the adolescents are reported to suffer from primary hypertension. This shift can be linked to a myriad of factors such as sedentary lifestyles, dietary changes, urbanization, excessive use of technology, obesity, high fat and calorie intake, smoking and alcohol consumption. According to the World Health Organization (WHO), the two primary strategies for proactive disease management are maintaining healthy eating habits and engaging in regular physical exercise [7]. The Center for Disease Control and Prevention recommends a minimum of 10,000 steps per day to enhance lifestyle. Insufficient physical activity contributes to elevated blood cholesterol, which leads to increase in blood pressure [8]. Regular exercise helps to reduce oxidative stress and enhance endothelial function. A study from Japan suggests that hypertensive patients should aim for 30 to 180 minutes of exercise daily [9]. The increased prevalence of hypertension among university students is attributed to modern dietary patterns, which are defined by diminished intake of dietary fiber, vegetables, and fruits and a higher consumption of salty, sugary, and high-fat foods. Studies propose that a junk food diet accelerates heart failure primarily through its effects on hypertension. Excessive calorie intake leads to obesity, which is strongly linked to increased blood pressure [10]. Elevated blood pressure contributes to heart failure by causing structural and functional changes in the heart. High glycemic and saturated fat rich foods exacerbate hypertension by increasing sympathetic activity and renal sodium retention, further elevating arterial pressure. These dietary factors also disrupt heart metabolism, leading to oxidative damage and cell apoptosis, which worsen heart function. Consequently, a high glycemic, high saturated fat diet not only aggravates hypertension but also accelerates the progression to heart failure [11]. Hence this study was conducted to identify university students who are asymptomatic for hypertension and assess their varied dietary and physical habits, along with any parental history.

## Materials and methods

Study design

A cross-sectional study was conducted on university students at a private university in North India.

Sample size

The sample size formula, [𝑛 = (4𝑝 (1 − 𝑝)/L2], was used to calculate the sample size. Based on a previous study [10], the prevalence of hypertension at 8% was used to get the maximum sample size by considering a 95% confidence interval and marginal error (𝑑) of 3%. The final sample size was determined to be 330.

Sampling method

For the study, a detailed list of students was obtained from the university authorities. The students who met the inclusion criteria were selected. They were explained about the nature of the study and informed consent was obtained from them. Details of the venue and time for measuring blood pressure were shared with the participants. The students who reported at the venue were included in the study, screened for blood pressure and asked to fill out a Google form containing questions to evaluate their lifestyle simultaneously.

Inclusion and exclusion criteria

The study included both undergraduate and postgraduate university students who met certain eligibility criteria such as being between 18 and 30 years of age and willing to participate in the study after signing an informed consent. Exclusion criteria included students with known diseases like hypertension, diabetes mellitus, bronchial asthma, or any cardiovascular disease.

Data collection

In the study, the respondents were provided with a pre-validated structured questionnaire comprising nine questions in four sections. Three questions were related to demographics and other questions were on parental hypertension history, current blood pressure measurements, and intake of Western fast food, sugar-sweetened beverages, high-fat foods, packaged snacks, meat products, and instant food along with the duration and intensity of exercise.

Evaluation and interpretation of blood pressure

Blood pressure was measured manually by trained medical personnel using a sphygmomanometer. The participants were asked to sit and relax for five minutes. Then the blood pressure was measured three times at an interval of two minutes between each reading. The average of the three readings was taken. If the values were less than 120 mmHg systolic and less than 80 mmHg diastolic, it was considered normal blood pressure. Prehypertension meant systolic pressure between 120 and 139 mmHg and diastolic pressure between 80 and 89 mmHg. Hypertension was defined as increased blood pressure, with a systolic value of 140 mmHg or above and a diastolic value of 90 mmHg or more, according to the JNC 8 guidelines [2].

Instrument used: Google forms in the English language

*Quality control*: A pre-validated questionnaire was taken from a previous study [5] and necessary modifications were made according to the present study. Then the questionnaire was pretested by experts in the field for content validity and internal consistency and Google forms were used to circulate the questions to the participants.

Proforma

The Google form containing the questions was shared with the participants, while the measurement of blood pressure was being done. The blood pressure readings were collected digitally in the same form.

*Consent and confidentiality:* The consent to participate in the study was taken in the beginning itself. The respondents were assured of the confidentiality of the survey by protection of the identity of the participants.

Statistical analysis

The data was coded and recorded in a Microsoft Office Excel sheet (Microsoft, Redmond, WA). The total data was presented as individual tables. Data was analyzed using IBM SPSS Statistics version 27.0 (IBM Corp., Armonk, NY). Descriptive statistics such as mean, standard deviation, and percentage were also used. The association between two categorical variables was explored using the chi-square test. Statistical significance was kept at p < 0.05.

## Results

A cross-sectional study was conducted among university students, with 102 male and 235 female participants. Most participants were in the 18-22 age group (N=169), and most were undergraduate students (N=260) (Table 1).

**Table 1:** Demographic distribution of the study population

Variable	Options	N	%
Gender	Males	102	30.3
	Females	235	69.7
Age group	18- 22 years	169	50.1
	23-26 years	144	42.7
	27-30 years	24	7.1
Education	Post-graduation	77	22.8
	Undergraduation	260	77.2
Total		337	100.0

The study classified the participants into three categories: normal, prehypertensive, and hypertensive. The data shows a clear gradient in systolic and diastolic blood pressure measurements across the different categories, from normal to hypertensive. Most of the participants had normal blood pressure, but the study observed an increasing trend of prehypertension among the students. The mean systolic blood pressure in prehypertensives was 124.6+6.81 mmHg and in hypertensives was 140.1+ 23.91 mmHg (Table 2). 

**Table 2:** Mean systolic and diastolic blood pressure of the study population BP: Blood pressure; SBP: systolic blood pressure; DBP: diastolic blood pressure

	N (%)	SBP	DBP
Normal BP	171 (50.7%)	107.8+7.08	68.5+6.58
Prehypertensive	124 (36.8%)	124.6+6.81	76.3+9.14
Hypertensive	42 (12.5%)	140.1+23.91	92.1+14.78
Total population	337 (100%)	118.04+15.52	74.3+11.71
p-value		0.001	0.001

Table 3 shows that there is a significant difference in the distribution of prehypertension and hypertension based on gender and age. Males were prone to developing prehypertension (54.9%) as compared to females (28.9%) and about 12.7% of the females had hypertension, which was statistically significant. Although women were more likely than men (11.7%) to have hypertension, prehypertension (28.9%) was more common than hypertension in females (12.7%). It's interesting to note that although 36% of participants were prehypertensive in the age group of 18-22 years, no significant association was found between the age groups of the students and the prevalence of prehypertension and hypertension.

**Table 3:** Distribution of the study population into normal, prehypertensive and hypertensive according to gender and age BP: Blood pressure

		N (%)			p-value
		Normal BP	Prehypertension	Hypertension	
Gender	Males	34 (33.3%)	56 (54.9%)	12 (11.7%%)	0.001
	Females	137 (58.3%)	68 (28.9%)	30 (12.7%)	
Age group	18- 22 years	80 (47.3%)	61 (36%)	28 (16.5%)	0.114
	23-26 years	81 (56.2%)	51 (35.4%)	12 (8.3%)	
	27-30 years	10 (41.6%)	12 (50%)	2 (8.3%)	

In the study, 124 participants who had blood pressure in the prehypertensive range had a parental history of hypertension. Participants whose parents have hypertension are more likely to be hypertensive themselves, suggesting a possible familial tendency (p=0.06), indicating that the association is not statistically significant, but there is a close potential trend or association that exists as depicted in Table 4.

**Table 4:** Comparison of groups with parental history of hypertension BP: Blood pressure

Groups	Does Parents have Hypertension		Total
	Yes	No	
Normal BP	65 (38%)	106 (62%)	171 (50.7%)
Prehypertensive	47 (37.9%)	77 (62.1%)	124 (36.8%)
Hypertensive	24 (57.1%)	18 (42.9%)	42 (12.5%)
p-value	0.060		

The participants' diets were evaluated using various dietary patterns. Table 5 shows that the participants who consumed sugared beverages (64.5%) and instant foods (43.2%) three to four days per week, along with high-fat food consumption (48%) and meat (57.1%) every day had a higher prevalence of prehypertension. In case of hypertension, consumption of high fat food (28%) and instant foods (25%) every day along with packaged food (17.6%) and Western fast food (16.2%) every three to four days in a week led to a higher prevalence. The consumption of sugary beverages and Western fast food showed a statistically significant relationship with increased blood pressure, while consumption of high-fat foods and instant foods also showed a close-to-significant relationship.

**Table 5:** Comparison of blood pressure readings in study groups with diet history BP: Blood pressure

		Normal BP	Prehypertensive	Hypertensive	p-value
Frequency consumption of sugared beverages	1-3 days/ month	63 (44.4%)	57 (40.1%)	22 (15.5%)	0.001
	1-2 days/week	31 (58.5%)	13 (24.5%)	9 (17%)	
	3-4 days/ week	11 (35.5%)	20 (64.5%)	0	
	5-6 days/ week	30 (83.3%)	4 (11.1%)	2 (5.6%)	
	Everyday	17 (41.5%)	18 (43.9%)	6 (14.6%)	
	None	19 (55.9%)	12 (35.3%)	3 (8.8%)	
Frequency consumption of high-fat food	1-3 days/ month	84 (53.2%)	51 (32.3%)	23 (14.6%)	0.054
	1-2 days/week	30 (53.6%)	20 (35.7%)	6 (10.7%)	
	3-4 days/ week	15 (51.7%)	13 (44.8%)	1 (3.4%)	
	5-6 days/ week	18 (47.4%)	15 (39.5%)	5 (13.2%)	
	Everyday	6 (24%)	12 (48%)	7 (28%)	
	None	18 (58.1%)	13 (41.9%)	0	
Frequency consumption of packaged snacks	1-3 days/ month	76 (53.9%)	46 (32.6%)	19 (13.5%)	0.199
	1-2 days/week	16 (35.6%)	23 (51.1%)	6 (13.3%)	
	3-4 days/ week	21 (61.8%)	7 (20.6%)	6 (17.6%)	
	5-6 days/ week	20 (51.3%)	15 (38.5%)	4 (10.3%)	
	Everyday	14 (60.9%)	7 (30.4%)	2 (8.7%)	
	None	24 (43.6%)	26 (47.3%)	5 (9.1%)	
Frequency consumption of meat	1-3 days/ month	19 (33.3%)	28 (49.1%)	10 (17.5%)	0.135
	1-2 days/week	33 (63.5%)	14 (26.9%)	5 (9.6%)	
	3-4 days/ week	7 (50%)	7 (50%)	0	
	5-6 days/ week	11 (55%)	7 (35%)	2 (10%)	
	Everyday	2 (28.6%)	4 (57.1%)	1 (14.3%)	
	None	99 (52.9%)	64 (34.2%)	24 (12.8%)	
Frequency consumption of instant food	1-3 days/ month	63 (51.6%)	42 (34.4%)	17 (13.9%)	0.091
	1-2 days/week	18 (40%)	16 (35.6%)	11 (24.4%)	
	3-4 days/ week	17 (45.9%)	16 (43.2%)	4 (10.8%)	
	5-6 days/ week	22 (64.7%)	9 (26.5%)	3 (8.8%)	
	Everyday	3 (75%)	0	1 (25%)	
	None	48 (50.5%)	41 (43.2%)	6 (6.3%)	
Frequency consumption of Western fast food	1-3 days/ month	83 (51.2%)	53 (32.7%)	26 (16%)	0.011
	1-2 days/week	16 (35.6%)	25 (55.6%)	4 (8.9%)	
	3-4 days/ week	14 (37.8%)	17 (45.9%)	6 (16.2%)	
	5-6 days/ week	16 (76.2%)	4 (19%)	1 (4.8%)	
	Everyday	8 (80%)	1 (10%)	1 (10%)	
	None	34 (54.8%)	24 (38.7%)	4 (6.5%)	

Table 6 shows that 51% of participants who did not indulge in regular exercise showed signs of prehypertension. On the other hand, 56.7% of the participants who indulged in regular brisk walking had normal blood pressure levels. This suggests a significant association between the frequency of exercise and the presence of prehypertension or normal blood pressure. Specifically, students who did not perform any physical exercise (10.5%) or performed exercise for less than 30 minutes (15.1%) had higher prevalence rates of hypertension compared to those who performed exercise for 30 minutes to 1 hour (9.9%). This indicates a statistically significant relation between exercise frequency (p value=0.002) and exercise duration (p value=0.007) with hypertension.

**Table 6:** Comparison of blood pressure readings in study groups with frequency, intensity and duration of exercise BP: Blood pressure

		Normal BP	Prehypertensive	Hypertensive	p-value
Exercise frequency	No regular exercise	41 (42.7%)	49 (51%)	6 (6.3%)	0.002
	Regular brisk walking	59 (56.7%)	34 (32.7%)	11 (10.6%)	
	Regular moderate	64 (55.2%)	31 (26.7%)	21 (18.1%)	
	Regular vigorous	7 (33.3%)	10 (47.6%)	4 (19%)	
Exercise duration	None	13 (34.2%)	21 (55.3%)	4 (10.5%)	0.007
	Less than 30 min	83 (54.6%)	46 (30.3%)	23 (15.1%)	
	30 min to 1 hour	39 (42.9%)	43 (47.3%)	9 (9.9%)	
	1-2 hours	9 (90%)	0	1 (10%)	
	More than 2 hrs	27 (58.7%)	14 (30.4%)	5 (10.9%)	

## Discussion

In the last two decades, there has been a surge in the reported cases of hypertension throughout the world, particularly in the younger age group [12]. This raises the need to outline the purpose and findings of a study investigating the prevalence of prehypertension and hypertension among university students. The study results show that 36.8% of the participants had prehypertension. This prevalence compares to similar studies from other universities: 30.7% in Bahrain, 27.1% in Palestine, and 23.3% in Jordan [11,13,14].

The prevalence of hypertension among the study participants was 12.5%, which is comparable to studies on Moroccan students, among whom the prevalence was 9.6% [15] and university students in Bahrain, among whom the prevalence was 8% [11]. In addition, research by Baig et al. [16] reported that, although the rates were lower than those reported in two studies conducted in Egypt (16.7% and 26.5%) [17], medical students in Jeddah and Kuwaiti college students had a prevalence of 9.3% and 11.8%, respectively [18]. The present study shows that age may not play a crucial role in the prevalence of prehypertension and hypertension among university students in this particular sample. However, a study conducted on university students in Bahrain observed that a higher incidence of blood pressure was seen among older male students [11]. The variation in the age spans of the research population under study, varying thresholds for measuring hypertension, sampling methods, study location, and study duration may further contribute to variation in prevalence rates of prehypertension among these studies.

Our study results show that males (54.9%) were more prone to developing prehypertension while females had more tendency to develop hypertension (12.7%). This is in contrast to a study conducted at Qassim University, where students showed the reverse trend: 44.9% of male students and 17.7% of female students had hypertension [19].

In our study, 124 college students had a positive family history of hypertension. While 37.9% of the prehypertensive students and 38% of the normotensive students had a family history of hypertension, 57.1% of the hypertensive students had hypertensive parents. These findings were consistent with other studies that demonstrated a significant familial connection to both prehypertension and hypertension [20,21].

Participants’ diet was evaluated using various dietary patterns in this study, and the data collected suggests that dietary patterns are closely associated with elevated blood pressure in asymptomatic young university students. A statistically significant correlation was found between the use of sugar-filled beverages (p=.001), Western fast food (p=0.011), and elevated blood pressure. Similar results were found in a study conducted by Xi et al., which showed an increased risk of hypertension was strongly associated with higher sweetened sugar beverage use [22]. Western fast food products contain an abundance of salt and fat, which has frequently been associated with high blood pressure. The salts used in these junk foods affect the renal system and alter the renin-angiotensin system, causing increased blood pressure. It further compromises the wellness of the heart, causes arterial stiffness, and left ventricular hypertrophy and leads to various cardiovascular diseases [23]. In addition, the Western fast food diet also contains increased levels of oxycholesterol, which promotes atherosclerosis, further leading to myocardial infarction. The WHO recommends limiting salt intake below 5 g per day to reduce the risk of hypertension [5]. Similar results were observed in a study conducted by Sundar et al. [24], which showed that more than 32% of high school students consume junk food regularly instead of the main meal, out of which 21.5% of the students suffer from hypertension.

In this study, prehypertension was seen in 51% of those students who did not exercise regularly. This highlights that physical inactivity is directly associated with the presence of prehypertension and increased blood pressure. These findings are in agreement with the study conducted by El-Agroudy et al. [11]. This is in contrast to the study conducted by Sundar et al. [24], which stated that physical inactivity was not significantly associated with hypertension in students.

Physical activity is an important lifestyle modification for the prevention and treatment of hypertension and similar results were seen in multiple studies [25-27]. Lack of physical activity is often linked to increased body weight, which can place additional strain on the heart and blood vessels. Excessive body fat contributes to heightened peripheral resistance, leading to elevated blood pressure. In contrast, regular physical activity enhances the heart’s efficiency in pumping blood, thereby lowering resting blood pressure and improving the capacity for vasodilation. Engaging in consistent exercise helps to reduce body fat, alleviate cardiovascular strain, and promote overall heart health [5]. It is essential to screen the asymptomatic, young adults for hypertension so that students at risk can be identified and adequate preventive and therapeutic measures can be undertaken.

Limitations of the study

The main limitation of the study was that it was conducted on students of a single university and therefore the results cannot be generalized. The results produced cannot be extrapolated to other regions that possess different demographic and socio-economic conditions. Secondly, the lack of long-term follow-up limits the ability to evaluate the persistence and long-term impact of the identified risk factors on the development of hypertension in young adults.

## Conclusions

The research findings reveal a high prevalence of hypertension and prehypertension among asymptomatic university students. The study also identifies key risk factors for increased blood pressure, including a positive family history of hypertension, male gender, increased consumption of fast food and sugary beverages and inadequate regular physical exercise among university students. These findings highlight that hypertension is a rising public health concern that needs to be addressed. It also emphasizes the significance of implementing targeted health education programs aimed at fostering healthy lifestyle practices among young adults.
